# The performance of domestic dogs (*Canis lupus familiaris)* on two versions of the object choice task

**DOI:** 10.1007/s10071-021-01500-9

**Published:** 2021-03-09

**Authors:** Hannah Clark, David A. Leavens

**Affiliations:** School of Psychology, University of Sussex, Falmer, BN1 9QH East Sussex UK

**Keywords:** Object choice task, Dogs, Comparative cognition, Domestication hypothesis

## Abstract

Object choice task (OCT) studies are widely used to assess the phylogenetic and ontogenetic distribution of the understanding of communicative cues, with this understanding serving as a proxy for the discernment of communicative intentions. Recent reviews have found systematic procedural and methodological differences in studies which compare performances across species on the OCT. One such difference concerns the spatial configuration of the test set-up, specifically the distances between the two containers (inter-object distance) and the subject–experimenter distance. Here, we tested dogs on two versions of the task: a central version in which the containers were in the subjects’ direct line of vision, and a peripheral version in which the position of the containers was distal to the subject. Half of the subjects were tested with a barrier in the testing environment (as nonhuman primates are tested) and the other half without. We found that dogs tested with a barrier performed significantly better in the central version and were more likely to fail to make a choice in the peripheral version. Dogs tested without a barrier showed comparable performance on the two versions. We thus failed to find support for the distraction hypothesis in dogs. We discuss potential explanations for this, highlighting how methodological differences in the presentation of the OCT can influence outcomes in studies using this paradigm.

Object choice task (OCT) studies are frequently used as evidence in support of theories that appeal to evolutionarily derived socio-cognitive competencies in the comprehension of deictic, referential gestures and the discernment of communicative intentions, in a range of vertebrate species (e.g., Povinelli et al. 1997, 1999; Tomasello et al. 1997). The OCT is designed to assess an individual’s ability to comprehend deictic cues, such as pointing, and involves an experimenter baiting one of two or more containers and then indicating the location of the hidden reward to the subject using a directive cue, such as pointing or direct gaze. Over multiple trials, above-chance performance in selecting the baited container constitutes evidence of subjects’ comprehension of the communicative cue. Interest in pointing comprehension abilities stems from gestural origin theories of language, which assert that spoken language evolved from complex gestural communication developed as an adaptive response to life on the savannah, after humans (*Homo sapiens*) separated from the rest of the hominid lineage (Arbib et al. 2008; Corballis 1999). Specific focus is given to declarative points, which are defined as points intended to direct attention (Bates et al. 1978), distinguished from imperative points, which function to obtain a desired object; declarative points have been linked to the emergence of joint attentional skills and language (Dawson et al. 2004). The relative abilities of nonhuman primates, therefore, in comprehending pointing cues have been widely studied, and their apparently poor performance (Herrmann et al. 2007; Itakura et al. 1999; Kirchhofer et al. 2012; Povinelli et al. 1997, 1999; Tomasello et al. 1997) has been used to suggest that the understanding of declarative points is, among primates, a human-unique ability and, further, that it entails a suite of socio-cognitive skills such as theory of mind (Baron-Cohen 1995), shared intentionality (Tomasello and Carpenter 2007) and cooperation (Moll and Tomasello 2007) that other primates do not possess.

In contrast, domestic dogs’ onsistently good performance on the OCT (e.g. Riedel et al. 2007; Viranyi et al. 2008) has been used to promote domestication theories, built on the premise that, through years of domestication, dogs have evolved specialised socio-cognitive skills which endow them with the ability to comprehend human gestural and other deictic cues (Hare and Tomasello 2005). Recent reviews draw into question both the domestication hypothesis and the claims for human uniqueness in declarative cue comprehension, asserting that a number of procedural confounds that are prevalent in the comparative literature prohibit group-to-species generalisations which form the evidence base for claims of species differences. For example, Lyn (2010) highlighted the importance of rearing history in apes to their understanding of human communicative conventions. Moreover, Lyn et al. (2010) demonstrated the importance of pre-experimental exposure to humans in the development of pointing comprehension, finding that enculturated apes, who have backgrounds rich in exposure to humans, display significantly higher success rates in point-following than institutionalised apes with less pre-experimental history with humans. Russell et al. (2011), whose ape samples were matched for age, sex and species, reported similar results, with the enculturated sample performing at similar levels to those reported for 2.5-year-old human children in previous studies. Similar effects have been found for dogs: those with less experience of human interaction than the typically tested pet dogs (e.g., kennel-raised or shelter dogs) demonstrate lower success rates on the OCT (D’Aniello et al. 2017; Lazarowski and Dorman 2015; Osborne and Mulcahy 2019; Udell et al. 2010a, b).

Whilst ontogenetic factors have been shown to affect performance on the OCT, Mulcahy and Hedge (2012) suggested that it may be methodological differences which better account for the ape-dog disparities in performance compared with phylogenetic explanations. They defined two versions of the OCT, the central version and the peripheral version. The central version involves the subject, experimenter and containers being positioned such that they are in close proximity, usually with subject and experimenter facing each other at a small table on which the containers are placed. In the peripheral version, in contrast, the subject and experimenter face each other at a distance of around 2 m, both equidistant to the containers which are placed on the floor, around 2 m apart. They outlined two ways in which the differential positioning of the testing apparatus may impact an individual’s performance. First, in the central version of the task, the subject has both the experimenter and the containers in their direct line of vision. Thus, the salience of the containers, one of which contains food (as the subject is aware), may distract the subject’s attention away from the cue being given. Second, in the peripheral version, the containers are placed at such a distance that the subject must locomote to the container to retrieve the hidden reward. Such extra effort required to obtain the hidden food may result in the subject paying increased attention to the cue being given by the experimenter. In a review of OCT studies, Mulcahy and Hedge (2012) argued that there is a tendency to test apes with the central version of the task, and dogs with the peripheral, and this was supported by Clark et al. (2019), who, in a review of 71 dog and ape OCT studies, found that dogs were tested with significantly greater inter-container distances than nonhuman primates. Mulcahy and Call (2009) and Mulcahy and Suddendorf (2011) both found increased performance by great apes when tested with a peripheral rather than central version of the task, providing support for Mulcahy and Call’s (2009) assertion that this methodological difference can affect individuals’ performance on the OCT. Similarly, Clark et al. (2019) found inter-object distance to be positively correlated with performance for both dogs and apes on a number of pointing cues on the OCT (differing in duration, laterality and distance, as outlined by Miklósi and Soproni 2006), such that as inter-object distance increased so did performance.

In the one study, to date, that has directly compared dogs’ performances with the two configurations of the OCT, Kraus et al. (2014) found that dogs performed above chance in both versions, but that performance was significantly lower in the central version of the task. Specifically, they found a success rate difference of 15% between the two conditions and they noted the similarity with Mulcahy and Call’s (2009) ape subjects whose performance in the two conditions differed by 17%. Kraus et al. (2014) therefore argued that their results provided support for the distraction hypothesis in dogs. Whilst Kraus et al. (2014) aimed to match their study design as closely as possible to that of Mulcahy and Call (2009), it differed in a number of ways, namely in the absence of test cages, the inter-object distances in the test set-up, and in the point type presented. With regard to the first of these differences, in Mulcahy and Call’s (2009) study, the apes were tested from within a test cage, thus imposing a barrier between the subject and the experimenter, and the subject and the containers. In Kraus et al’s (2014) study, dogs were not tested within test cages, a systematic cross-species confound in testing environment that Leavens et al. (2019) and Clark et al. (2019) note is prevalent in much of the comparative OCT literature. Clark and Leavens (2019) and Kirchhofer et al. (2012) found that the imposition of a barrier into an OCT protocol can have a detrimental effect on dogs’ performances.

With regard to the configuration of the test setup, the two studies differed in that Kraus et al. (2014) used a peripheral inter-object distance of 140 cm and a central inter-object distance of 45 cm, whereas Mulcahy and Call (2009) used distances of 250 cm and 60 cm, respectively. This is a difference of more than a metre in the peripheral version, an important factor to consider given previously mentioned findings that an increase in inter-object distance was associated with an increase in performance on the OCT (Clark et al. 2019).

Finally, Kraus et al. (2014) presented subjects with an ipsilateral momentary proximal point in both conditions, which involves presenting the pointing cue for three seconds before retracting the hand (as per Miklósi and Soproni 2006), with the distance between the experimenter’s finger and the container being around 20 cm in the central condition and 30 cm in the peripheral condition. In comparison, Mulcahy and Call (2009) used a cross-lateral dynamic pointing cue, which involves pointing across the body with the hand contralateral to the correct container and maintaining the position until the subject makes a choice. The distance between the experimenter’s finger and the container was approximately 100 cm in the peripheral condition (a distal point according to Miklósi and Soproni 2006) and 40 cm in the central condition (a proximal point). Miklósi and Soproni (2006) and Udell et al. (2013) have shown that the laterality, duration of presentation, and the finger-tip-to-container distance can all differentially affect performance on the OCT.

Given that the use of barriers in the testing environment in the form of cages, inter-object distance, and point type have all been shown to differentially affect an individual’s performance, direct comparison between Kraus et al.’s (2014) dogs and Mulcahy and Call’s (2009) apes is subject to the effects of a number of confounds in the test setups. In the current study, therefore, we aimed to replicate as closely as possible the testing conditions used by Mulcahy and Call (2009) with a sample of pet dogs, to investigate further whether differences in spatial configuration might affect the behavioural responses of dogs on the OCT, with the additional manipulation of the presence of a barrier between dogs and targets. We matched the spatial configuration and the point cue presented with those of Mulcahy and Call (2009), and half of the dogs were tested within a child’s playpen, designed to emulate a cage, following Clark and Leavens (2019). For control purposes, we also tested half of the dogs without the playpen. This would allow us to distinguish between the effects of the two manipulations: to ascertain the effects of the two configurations and effects of the imposition of the barrier. As such, the study used a 2 × 2 design, with configuration as a within-subjects variable and barrier as a between-subjects variable. We expect, following Mulcahy and Call’s (2009) and Kraus et al.’s (2014) findings, the dogs’ performance to be lower in the central version of the task than in the peripheral version, and that performance in both conditions will be lower in the presence of a barrier, in line with Clark and Leavens (2019) and Kirchofer et al. (2012).

## Methods

### Subjects

Twenty-six pet dogs (14 male, 12 female) took part in the study. Dogs ranged in age from four months to 12 years (*M* = 4.0 years, *SD* = 3.1) and comprised a variety of breeds (see Table 1 for individual subject and performance data). Dogs were recruited through advertisements placed on social media and face-to-face recruitment in a local park.

**Table 1:** Subject and performance data

Name	Breed	Sex	Age (years)	Barrier	First condition	Central trials correct	Peripheral trials correct
Hendrix	Shih Tzu x Chihuahua	M	7	Barrier	Central	1	0
Evie	Lurcher	F	3	Barrier	Central	4	2
Abbie	Cavalier King Charles Spaniel	F	0.75	Barrier	Central	2	2
Theo	Cocker Spaniel × Poodle	M	4	Barrier	Peripheral	4	4
Marnie	Yorkshire Terrier × Jack Russell	F	4	Barrier	Peripheral	4	1
Bruce	Manchester Terrier × Jack Russell	M	6	Barrier	Central	4	4
Dudley	Springer Spaniel	M	12	Barrier	Peripheral	4	2
Stanley	Cocker Spaniel × Poodle	M	0.33	Barrier	Central	4	1
Olly	Cocker Spaniel	M	0.83	Barrier	Peripheral	3	0
Eva	Red Fox Labrador	F	6	Barrier	Peripheral	3	4
Axie	Mongrel	F	4	Barrier	Peripheral	3	2
Bob	Jack Russell	M	4	Barrier	Peripheral	2	0
Jake	Springer Spaniel × Poodle	M	1	No Barrier	Peripheral	4	4
Gary	English Mastiff	M	2	No Barrier	Peripheral	3	4
Floki	Border Collie	F	3	No Barrier	Peripheral	3	3
Leyla	Cavalier King Charles Spaniel × Poodle	F	4	No Barrier	Central	0	3
Watson	Border Collie	M	5	No Barrier	Central	4	4
Tallulah	Jack Russell × Poodle	F	3	No Barrier	Peripheral	3	4
Bruce	Border Collie	M	12	No Barrier	Peripheral	2	3
Tilly	Border Collie	F	1	No Barrier	Central	2	0
Ruby	Jack Russell	M	4	No Barrier	Peripheral	1	2
Elvis	Cocker Spaniel	M	7	No Barrier	Central	4	4
Cookie	French Bulldog × Pug	F	1	No Barrier	Central	3	2
Penny	Boston Terrier	F	2	No Barrier	Central	4	2

None of the dogs had ever taken part in any studies before; however, given that all of the subjects were pets, and thus exposed to rich human interaction on a daily basis, it is likely that all subjects were somewhat familiar with pointing gestures. The extent of individual subjects’ familiarity with such cues or indeed with human interaction was a factor we were unable to control for. Subjects were tested individually by an unfamiliar experimenter, and testing took place in a local community hall. Subjects were randomly assigned to conditions prior to testing. Two dogs were excluded from the final analysis; one because she failed to complete testing due to being apparently highly anxious within the playpen, and the other because the video-recording of the test session was lost due to a technical error.

### Materials and setup

**Fig. 1:**
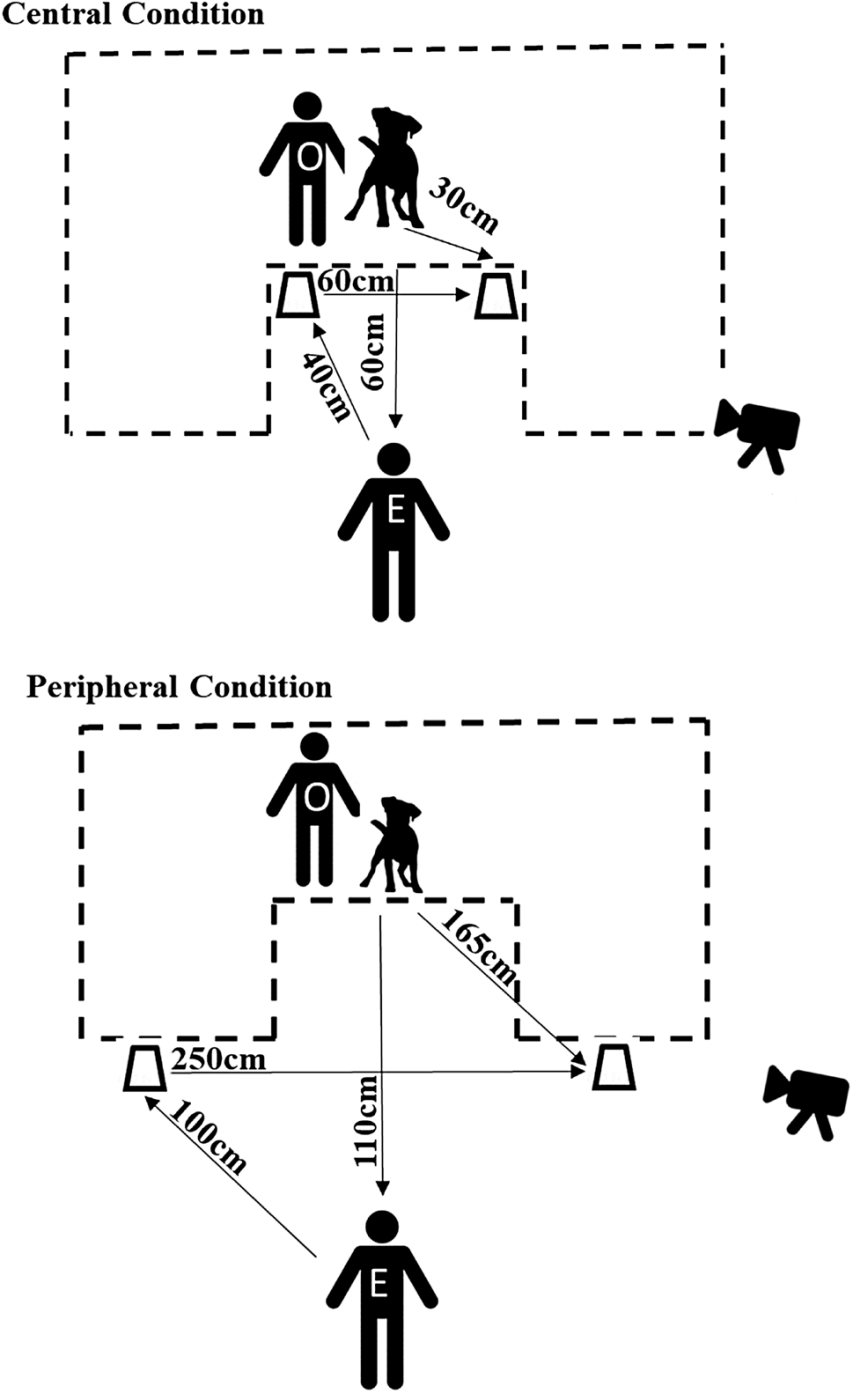
The configurational set-up of the central and the peripheral conditions. O = Owner; E = experimenter. Dashed line indicates playpen. Figure not to scale, distances involving agents are approximate

The playpens used in the barrier condition were two Dreambaby Royal Converta 3-in-1 Playpen Gates, measuring 380 × 4 × 74 cm (Dreambaby, Unit 53, Rosyth Business Centre, 16 Cromarty Campus, Rosyth, KY11 2WX, Scotland). The containers used to hide the bait were two opaque plastic cups. A premium commercial dry dog food was used for baiting the cups. All dogs were tested on a 1-m-long lead. All testing sessions were recorded on a Sony Handycam HDR-PJ410 video-camera (Sony, 1–7-1 Konan Minato-ku, Tokyo, 108–0075 Japan). In the central version of the task, the experimenter knelt at a distance of approximately 60 cm from the subject and the distance between the two containers was 60 cm; in the peripheral version, the subject–experimenter distance was approximately 110 cm and the inter-object distance was 250 cm (see Fig. 1). Because we exactly matched the relevant distances to those in Mulcahy and Call (2009), therefore, by the Pythagorean theorem, all angles between elements that were under experimental control would be identical to those in their study.

## Procedure

### Pre-test

On arrival at the hall, dogs were given time to explore the room, off lead, and the experimenter interacted with the dog and offered them treats, for them to become familiar with both the testing environment and the experimenter. The experimenter then showed the dogs the cups, allowing them to sniff them, and then, in sight of the dog, hid a treat under one of the cups, then showed it to the dog and gave it to them. This was repeated twice to show the dog that the experimenter had treats and that these could be found under the test cups.

### Test

The owner was then asked to put the dog on a 1 m lead and to stand with the subject on a marked point in the test room (this was inside a playpen for dogs tested with a barrier; for those tested without, it was the same location in the room but without the barrier present—see Fig. 1). The experimenter then baited both cups in view of the dog, saying “*name*… look” as she hid a treat under each cup. The order of baiting was counterbalanced across trials. She then returned to a point equidistant between the two cups, called the dog’s name and established eye contact, then pointed to one of the cups using a cross-lateral, dynamic pointing cue. In the central version, the distance between the experimenter’s finger and the cup was approximately 40 cm; in the peripheral version, it was approximately 100 cm (Fig. 1). The owner held the subject on the lead while the baiting took place and released the lead when the experimenter pointed. The experimenter maintained the pointing cue until either the subject made a choice (described below) or the trial timed out. If the subject made a correct choice, the experimenter gave the subject the piece of food (if they had not already retrieved it). Both dogs tested from within the playpen and those tested without were able to retrieve the food reward—the gap between the bars was such that dogs were able to either insert their snout and lift the cup or reach through with the paw to knock the container over and retrieve the reward. If the subject made an incorrect choice, both pieces of food were removed from the containers and the trial ended. If the subject failed to make a choice, the trial ended after one minute. The beginning of the trial was counted from when the pointing cue was presented and the subject was released to make a choice (after Udell et al. 2010b). Subjects were given four trials per condition, with the order of conditions counterbalanced prior to testing. The cued (correct) container was on the left or the right an equal number of times and the order was pseudorandomised such that it never appeared on the same side for more than two consecutive trials.

### Data scoring

All testing sessions were video recorded and coded at a later date. Trials were coded for correct/incorrect response and response latency. Following Udell et al.’s (2010b) recommendations, a correct response was categorised as one in which the subject first touched or came within 10 cm with their snout of the correct container. Any other response was categorised as incorrect. Incorrect responses were further categorised into incorrect choices, in which the subject first touched or came within 10 cm with their snout of the incorrect cup, and no choice, in which the subject failed to choose one of the cups before the end of 1 min.

### Reliability

All trials were coded by the first author, and a second coder, who was blind to the purposes of the study, coded a random sample of 20% of subjects’ testing sessions; that is, five dogs’ testing sessions constituting 40 trials. For correct choices, there was 100% agreement between the two coders, ĸ = 1.00, *p* < 0.001. There was excellent agreement for whether or not the subject made a choice, *ĸ* = 0.81, *p* < 0.001, and for response latency, *r*_s_ = 0.99, *p* < 0.001.

### Data analyses

Due to a lack of normal distribution in the data, all analyses used nonparametric tests. Binomial tests were used to test for above-chance performance, Wilcoxon signed-ranks test was used for the within-subjects comparisons and Mann–Whitney *U* tests were used for the between-subjects comparisons.

## Results

### Age and sex

We found no relationship between age and performance (Spearman’s *rho* = -0.04, *N* = 24, *p* = 0.852). In addition, we found no relationship between dogs’ sex and performance (Mann–Whitney *U* = 51.00, *p* = 0.210). Therefore, we do not further consider age or sex.

### Dogs tested with a barrier

#### Correct choices

**Fig. 2:**
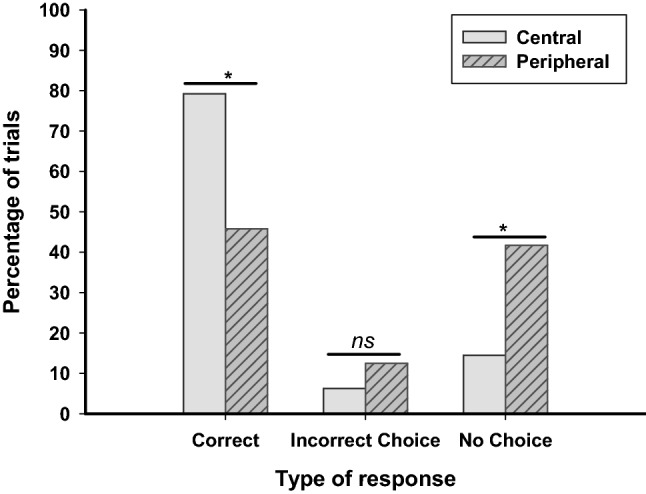
The percentage of trials in which dogs tested with a barrier made a correct choice, an incorrect choice and no choice in the central and peripheral versions of the task. Total number of trials per condition = 48. *Significant at *p* < .05

As a group, dogs performed above chance in the central version of the task, (binomial test, *p* < 0.001) but not in the peripheral version (binomial test, *p* = 0.665). Dogs chose the correct container on a significantly higher proportion of trials in the central version (*Mdn* = 0.88) than in the peripheral version (*Mdn* = 0.50) of the task, *Z* = − 2.46, *p* = 0.014. This shows that dogs tested with a barrier were more accurate in their responses in the central version than in the peripheral version. Figure 2 shows the percentage of trials on which the dogs tested with a barrier (a) chose the correct cup, (b) chose the incorrect cup, and (c) failed to make a choice.

### Incorrect choice and no choice responses

There was no significant difference in the proportion of responses that were incorrect choices between the central version (*Mdn* = 0.00) and the peripheral version (*Mdn* = 0.00) of the task, *Z* = − 1.00, *p* = 0.317. The proportion of trials on which the dogs failed to make a choice was significantly lower in the central version (*Mdn* = 0.00) than in the peripheral version (*Mdn* = 0.50), *Z* = − 2.27, *p* = 0.023. This shows that the dogs were similarly likely to make an incorrect choice in the two versions of the task, but that they were more likely to fail to respond when the cups were placed further apart.

### Dogs tested without a barrier

#### Correct choices

**Fig. 3:**
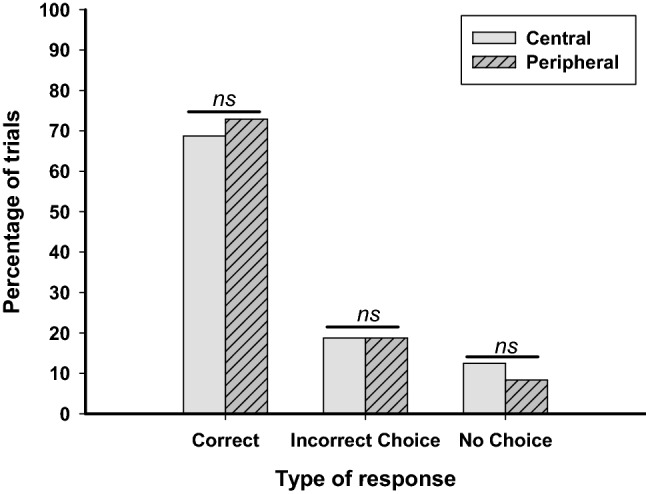
The percentage of trials in which dogs tested without a barrier made a correct choice, an incorrect choice and no choice in the central and peripheral versions of the task. Total number of trials per condition = 48. *Significant at *p* < .05

As a group, the dogs performed above chance in both the central version (binomial test, *p* = 0.002) and the peripheral version (binomial test, *p* = 0.013) of the task. There was no significant difference in the proportion of trials on which the dogs chose the correct cup between the central version (*Mdn* = 0.75) and the peripheral version (*Mdn* = 0.75) of the task, *Z* = −0.29, *p* = 0.774. This shows the dogs tested without a barrier performed equally well on both versions of the task. Figure 3 shows the percentage of trials on which dogs tested without a barrier (a) chose the correct cup, (b) chose the incorrect cup, and (c) failed to make a choice.

### Incorrect and no choice responses

There was no significant difference between the proportion of trials on which the dogs made an incorrect choice between the central (*Mdn* = 0.25) and the peripheral version (*Mdn* = 0.13), *Z* = -−0.14, *p* = 0.890. There was also no significant difference between the proportion of trials in which the dogs failed to make a choice between the central (*Mdn* = 0.00) and the peripheral version (*Mdn* = 0.00), *Z* = -−378, *p* = 0.705. This shows that there was no difference between the type of incorrect responses the dogs exhibited between the central and peripheral versions of the task, in the absence of a barrier.

## Barrier vs. no barrier comparisons

### Correct choices

On the central version of the task, there was no significant difference in the proportion of trials in which the dogs chose the correct cup between those tested with a barrier (*Mdn* = 0.75) and those tested without (*Mdn* = 0.88), Mann–Whitney *U* = 58.5, *p* = 0.411. Nor was there a significant difference between those tested with a barrier (*Mdn* = 0.50) and those tested without (*Mdn* = 0.75) on the peripheral version, Mann–Whitney *U* = 42.00, *p* = 0.073. This shows that the dogs tested with and without a barrier chose the correct cups on a comparable number of trials in the two versions of the task.

### Incorrect choice and no choice responses

There was no significant difference in the proportion of trials in which dogs made incorrect choices in the central version of the task between dogs tested with a barrier (*Mdn* = 0.00) and dogs tested without a barrier (*Mdn* = 0.25), Mann–Whitney *U* = 45.00, *p* = 0.075. There was also no difference in the proportion of incorrect choices on the peripheral version between dogs tested with a barrier (*Mdn* = 0.00) and dogs tested without (*Mdn* = 0.13), Mann–Whitney *U* = 60.00, *p* = 0.434. On the central version of the task, there was no difference in the proportion of trials on which the dogs failed to make a choice between dogs tested with a barrier (*Mdn* = 0.00) and dogs tested without a barrier (*Mdn* = 0.00), Mann–Whitney *U* = 67.00, *p* = 0.719. On the peripheral version of the task, dogs tested with a barrier failed to make a choice on a significantly higher proportion of trials (*Mdn* = 0.50) than dogs tested without a barrier (*Mdn* = 0.00), Mann–Whitney *U* = -−2.41, *p* = 0.016. This shows that the dogs tested with and without a barrier exhibited comparable types of incorrect responses on the central version of the task, but that dogs tested with a barrier showed an increased tendency to fail to make a choice on the peripheral version.

### Order of administration

For dogs tested with a barrier, there were no effects of order of administration on the proportion of correct choices in the central version (central first *Mdn* = 1.00; peripheral first *Mdn* = 0.75), Mann–Whitney *U* = 17.00, *p* = 0.930, nor in the peripheral version (central first *Mdn* = 0.50; peripheral first *Mdn* = 0.50), Mann–Whitney *U* = 17.50, *p* = 1.00.

For dogs tested without a barrier, there were no effects of order of administration on the proportion of correct choices in the central version (central first *Mdn* = 0.88, peripheral first *Mdn* = 0.75), Mann–Whitney *U* = 14.50, *p* = 0.560, nor in the peripheral version (central first *Mdn* = 0.63; peripheral first *Mdn* = 0.88), Mann–Whitney *U* = 12.00, *p* = 0.312. This shows that there were no order of administration effects for dogs tested with or without a barrier.

### Trial by trial analyses

For dogs tested with a barrier, there was no difference in the number of correct choices across trials in the central version, Cochran’s *Q* = 3.00, *p* = 0.392, nor in the peripheral version, Cochran’s *Q* = 0.55, *p* = 0.909.

For dogs tested without a barrier, there was no difference in the number of correct choices across trials in the central version, Cochran’s *Q* = 1.44, *p* = 0.697, or in the peripheral version, Cochran’s *Q* = 6.14, *p* = 0.105. This shows that both for dogs tested with and dogs tested without a barrier, there was no effect of successive administrations on their tendency to choose the correct container.

## Discussion

We tested dogs on a central and peripheral version of the OCT, attempting to replicate as closely as possible Mulcahy and Call’s configurational manipulations in their study of 19 juvenile and adult apes (comprised of chimpanzees, bonobos and an orangutan). Here, we failed to replicate their results with regard to configurational effects, finding that dogs tested with a barrier (akin to the apes tested in cages in Mulcahy and Call’s study) only performed above chance in the central version of the task, in which performance was significantly higher than in the peripheral version, and no effects of configuration for those tested without a barrier, with dogs performing above chance in both configurational conditions. This contrasts both with Mulcahy and Call’s (2009) study with apes and with Kraus et al.’s (2014) study with dogs, in which apes and dogs, respectively, demonstrated increased performance in the peripheral version of the task. Whilst our aim in the current study, which was also the aim of Kraus et al. (2014), was to replicate as closely as possible Mulcahy and Call’s (2009) testing conditions, there remain some differences in methodology between the three studies, which could serve as explanations for the lack of replication of the configurational effects. First, the scoring procedures used with apes and dogs differ as a function of species-specific capabilities. With their ape subjects, Mulcahy and Call (2009) scored a correct choice when the subject inserted their finger through a hole in the plexiglass window to touch the container, whereas in both our study and Kraus et al.’s (2014) study, scoring procedures followed those of Udell et al. (2010a, b) in which a correct choice was deemed to be such when a subject first touched or came within 10 cm of the container. Given that in both of the dog studies the same scoring procedure was used, and that Kraus et al. (2014), using this scoring procedure, replicated the configurational effects found by Mulcahy and Call (2009), we do not think that this difference in scoring procedure between the present study and that of Mulcahy and Call explains our failure to replicate their results.

A further difference between the studies is the reward that was offered to subjects upon selecting the correct container—Mulcahy and Call (2009) used a flattened grape, Kraus et al. (2014, p. 174) used “small pieces of commercial dog treat”, and in the current study, we used a premium commercial dry dog food. It may be that subjects were differentially motivated to work for the different foodstuffs offered, however, given that in all three studies, subjects selected a container significantly more than they failed to make a choice, we argue that subjects were motivated to work for the foods on offer.

A third difference between the studies is the baiting procedure used in each. In the current study, the experimenter baited both cups in view of the subject, then presented a pointing cue from an equidistant point. This differs from both Mulcahy and Call’s (2009) and Kraus et al.’s (2014) baiting procedures. In the former, one container was baited in view of the subject, placed behind an occluder, followed by the second empty container being shown to the subject, and then also being placed behind the occluder, before the experimenter either switched or pretended to switch the position of the containers. In Kraus et al.’s (2014) study, the experimenter baited the two containers “invisibly for test subjects” (p. 175) covering the baiting process with her body, then turned and simultaneously placed both pots on the test table. Thus, the studies differ both in terms of the number of containers which were actually baited and the visibility of the baiting procedure for the subjects. This could be a possible explanation for why we failed to replicate the distraction effects of the central configuration, however, given that subjects viewed both containers being baited in the current study, it could be argued that having the containers at a closer proximity would thus then be more distracting—two pieces of food easily within reach from which the subject must avert their attention to attend to the cue, rather than one. We would expect then, if dogs were being distracted by the salience of the containers, to see an increase in incorrect responses in the central condition when both containers were baited in full view, but in the current study, we found that, in fact, correct choices were more prevalent. An alternative explanation is that the presence of two pieces of food heightened the dogs’ focus on the cue—the salience of the food reward may have been increased due to there being two pieces and therefore dogs may have been more motivated to work for the reward. This possibility is one that could be explored in future studies using an alternative baiting method in which either baiting is occluded or there is no food reward in either of the containers.

A further difference in the methodologies used in the studies is in the test set-up, specifically the use of raised platforms to present the stimuli. In Mulcahy and Call’s (2009) study, ape subjects were tested sitting at a table, whereas in the studies with dogs, Kraus et al. (2014) placed the containers on two raised platforms (stacked boxes), and in the current study, the containers were placed on the floor. This means that the containers in the current study were more easily within reach of most of the subjects, and thus, we would expect the central version to be more distracting, which again contrasts with our findings.

Furthermore, the dogs in the current study were tested in the presence of their owner, contrasting with Mulcahy and Call’s (2009) subjects who were tested individually. Although the presence of the owner could potentially influence the dogs’ behaviour, we do not think that this can explain the difference in findings reported between our study and those of Mulcahy and Call (2009) and Kraus et al. (2014) because the dogs tested by Kraus et al. (2014) were also tested in the presence of the owner, and also because previous studies have found the presence of the owner, and even inadvertent cuing, to have little effect on dogs’ performance on the OCT (Hegedüs et al 2013; Schmidjell et al 2012).

In addition, the number of trials presented to subjects differs between the three studies—Mulcahy and Call’s (2009) ape subjects completed 24 trials per condition, Kraus et al.'s (2014) dog subjects completed 12 trials per condition, and, in the current study, subjects completed four trials per condition. This was following Clark and Leavens (2019) who found significant differences in dogs' behaviour and performance on an OCT when comparing dogs tested with and without a barrier using four trials per condition also. Whilst testing subjects on a greater number of trials per condition would result in more data on which to base our analyses, given that Clark and Leavens (2019), and in the current study, our trial-by-trial analyses showed no differences across trials in dogs’ responses, we do not believe this would have a substantial impact on our findings.

Finally, the studies differ in the type of pointing cue presented to subjects. In the current study, and in Mulcahy and Call (2009), a cross-lateral, dynamic pointing cue was used, meaning the cue was in place until a subject made a choice, or the trial timed out, whereas Kraus et al. (2014) presented their subjects with an ipsilateral, momentary proximal pointing cue, meaning subjects were presented with the cue for just three seconds. As Miklósi and Soproni (2006) highlight, momentary and dynamic cues differ in terms of the memory demands on subjects—with a momentary cue, the subject must attend to and remember the direction of the cue, whereas with a dynamic cue, no such memory demands exist. It may be, then, that dogs presented with a momentary cue are more likely to be subject to distraction if the containers are in their direct line of vision, because not only must they attend to the cue over the highly salient containers, they must also remember the direction of the cue and use this to inform their response.

When comparing dogs tested with and without a barrier, we found no significant differences in performance across the two versions of the task. This was surprising, given that Kirchhofer et al. (2012) and Clark and Leavens (2019) found suppressive effects on performance associated with the imposition of a barrier. On the central version of the task, dogs tested with a barrier actually had a higher success rate, choosing the correct cup on 75% of trials, than those tested without, who had a success rate of 69%. One explanation for this is that the barrier perhaps reduces dogs’ susceptibility to distraction—having the barrier between the subject and the containers may decrease the salience of the cups and promote attention to the cue. In contrast, in the peripheral version of the task, although there was no significant difference, there was a trend towards poorer performance associated with the presence of a barrier, and dogs both failed to perform above chance in the barrier condition and failed to make a choice on a significantly greater number of trials. This echoes the findings of Clark and Leavens (2019) who found a similar increase in no choice responses when dogs were tested with a barrier. Clark and Leavens (2019) suggested that this may be explained by the Referential Problem Space (Leavens 2021; Leavens et al. 2005), that is, the barrier may increase perceptions of the reward as being unobtainable (even though, it is, in fact obtainable). This perception coupled with the extra effort associated with obtaining a reward which is placed far away from the subject in the peripheral condition may explain, in the current study, why the dogs tested with a barrier failed to make a choice on 42% of peripheral trials. A related potential future avenue for investigation would be to compare the performance of dogs raised in alternative environments to pet dogs, specifically shelter or kennel dogs, who arguably would have more experience with barriers (in the form of cages). Whilst previous research shows that such groups typically perform more poorly on point-following tasks than pet dogs (e.g. Lazarowksi and Dorman 2015; Osborne and Mulcahy 2019; Udell et al. 2010a, b), it would be of interest to see what effects, if any, the imposition of a barrier in the testing environment would have on their behaviour and performance. Another possible factor to consider is the visual acuity of the individual and the extent to which they may be able to see either the structure of the barrier (be that bars or wire mesh) and the objects behind it, with this being influenced by the distance between the subject and the apparatus as well as variables such as age and species [see Bard et al. (1995) for a discussion of the development of visual acuity in chimpanzees, and Miller and Murphy (1995), for a discussion of visual acuity in dogs].

There are some limitations to the current research, the first of which is our modest sample size of 24 subjects. Although a sample size greater than this may be preferable, here our objective was to investigate the generalisability of the configuration effects found by Mulcahy and Call (2009) whose sample consisted of 19 subjects. Given that Mulcahy and Call (2009) found an effect with this size sample, we think that our use of 24 subjects is comparable in power to that of the original study. Our intention in this study was not to demonstrate a species proclivity but to investigate the effect of an experimental manipulation on a sample of comparable size, but a different species, to that studied by Mulcahy and Call (2009). We do, however, acknowledge that an interesting future direction would be to study the effect of this manipulation with a greater sample of dogs.

In addition, as an anonymous reviewer helpfully pointed out, the dogs tested were of a wide range of ages, thus representing various life history stages. In their original study, Mulcahy and Call (2009) found an effect of configuration on performance on the OCT with a sample of apes which included both juveniles and adults (specific ages were not specified). We, therefore, reasoned that if this effect of configuration generalises to dogs, we should be able to find a comparable effect with a comparable sample, which also includes both juvenile and adult subjects. Furthermore, our sampling of dogs is in line with Dorey, Udell and Wynne (2010) and Udell et al.'s (2013) assertions that there is no detriment to performance associated with age for dogs aged four months and over. It is consistent with age sampling in other dog OCT studies in the literature (e.g. Clark and Leavens 2019; Hare and Tomasello 1999; Udell, Dorey and Wynne 2008; Udell et al. 2010a, b). Notwithstanding, we do acknowledge that this is a legitimate point which warrants further investigation. As noted by Leavens et al. (2019), a lack of consideration of a subject’s life history stage when comparing across species is a prevailing issue in the animal cognition literature, and it may well be that puppies and seniors perform in systematically different ways to adult dogs. Whilst our objective in the current study was to investigate the generalisability of the configurational effects found by Mulcahy and Call (2009), and therefore, our sampling was consistent with their ape sampling, we recognise that an interesting future direction would be to investigate the potential effects of life-history stage.

A final limitation of the current study is our baiting method, specifically the choice to bait both containers prior to presenting the cue. This method was used to control for possible olfactory cues associated with baiting one container only. A drawback of this method is that some dogs were able to retrieve the food reward even when an incorrect choice was made. We do not believe, however, that this has had a substantial effect on our results, first because dogs tested without a barrier, and thus with easier access to the containers to retrieve the rewards, performed above chance in both the central and peripheral versions of the task demonstrating that they were attentive and responsive to the cue being given. However, we recognise that this method of baiting may have influenced dogs’ behaviour, and as such we would recommend a method more akin to that used by Udell et al. (2013) in which food was not contained within or on the containers used until the subject made a choice. This allows olfactory cues to be controlled for whilst also eliminating the possibility of inadvertent reinforcement of incorrect choices.

We did not find support for the distraction hypothesis in dogs tested either with or without a barrier, for the former, in fact, the central version facilitated performance, and for the latter, that performance was comparable. This contrasts with Kraus et al.’s (2014) findings and further highlights the effects that cue types may have on performance, as noted by Miklósi and Soproni (2006) and Udell et al. (2013). Indeed, an interesting future direction would be to further investigate how configuration and cue type may interact to affect subjects’ performance. This could be done by testing dogs across a range of cue types differing in their distance and temporal properties, similar to Udell et al. (2013), using both a central and a peripheral configuration. We found the presence of a barrier to affect behavioural responses in the peripheral version, and a statistically non-significant trend towards this finding in the central version which echoes Clark and Leavens (2019) findings in which greater sample sizes were used.

In conclusion, here we found a complex interaction between two environmental influences on performance in dogs, with responses to central and peripheral versions of the OCT differing as a function of the presence of a barrier. This builds on previous studies (e.g. Clark and Leavens 2019; Clark et al. 2019, 2020; Miklósi and Soproni 2006; Mulcahy and Hedge 2012; Mulcahy and Suddendorf 2011; Udell et al. 2013) showing that methodological differences in the presentation of the OCT can impact on individual performance and behaviour, and shows the importance of ensuring comparable testing conditions before generalising from individuals to a species as a group, and before making cross-species comparisons. This study adds to the growing OCT literature which emphasises the necessity of addressing systematic methodological confounds prior to speculating about the evolutionary roots of socio-cognitive skills, based on apparent species difference in performance. Without consideration of the effects of these confounding variables, it is premature to attribute subjects’ responses to their selective histories.

## Declarations

### Availability of data and material (data transparency)

Data are available on request from the corresponding author.
